# The Presence of *Leishmania braziliensis* DNA in the Nasal Mucosa of Cutaneous Leishmaniasis Patients and the Search for Possible Clinical and Immunological Patterns of Disease Progression: A Cross Sectional Study

**DOI:** 10.3389/fcimb.2021.744163

**Published:** 2021-10-14

**Authors:** Daniel Holanda Barroso, Otávio de Toledo Nóbrega, Carla Nunes de Araújo, Gustavo Subtil Magalhães Freire, Sofia Sales Martins, Bruna Côrtes Rodrigues, Ciro Martins Gomes, Raimunda Nonata Ribeiro Sampaio

**Affiliations:** ^1^ Programa de Pós-Graduação em Ciências Médicas, Faculdade de Medicina, Universidade de Brasília (UnB), Brasília, Brazil; ^2^ Hospital Universitário de Brasília, Universidade de Brasília, Brasília, Brazil; ^3^ Laboratório de Dermatomicologia da Faculdade de Medicina, Universidade de Brasília, Brasília, Brazil; ^4^ Pós-Graduação de Ciências da Saúde da Faculdade de Ciências Saúde, Universidade de Brasília, Brasília, Brazil; ^5^ Programa de Pós-Graduação em Medicina Tropical, Faculdade de Medicina, Universidade de Brasília (UnB), Brasília, Brazil

**Keywords:** *leishmaniasis*, immunology, multivariate analysis, mucocutaneous, flow cytometry

## Abstract

*Leishmania braziliensis* is the most important causal agent of American tegumentary leishmaniasis (ATL), and 3 to 5% of patients develop mucosal lesions. The mechanisms related to parasite and host immune interactions and the parasite life cycle that lead to dissemination to the mucosa are poorly understood. We aimed to detect *L. braziliensis* DNA in the nasal mucosa of cutaneous leishmaniasis (CL) patients with early mucous dissemination and to relate those findings to specific inflammatory responses. Nasal swabs were collected from patients with the cutaneous form of ATL. *L. braziliensis* DNA was investigated using TaqMan-based real-time PCR. The levels of serum cytokines (IL-12, IL-6, TNF-α, IL-10, IL-1β and IL-8) were measured by a multiplex cytometric array. A Poisson regression model was used to test prevalence ratios (PRs) and multivariate interactions of clinical and laboratory characteristics. Of the 79 CL patients, 24 (30%) had *L. braziliensis* DNA in the nasal mucosa. In the multivariate model, parasite DNA presence in mucosa was associated with a reduction in IL-12 levels (PR = 0.440; p=0.034), increased IL-6 levels (PR = 1.001; p=0.002) and a higher number of affected body segments (PR = 1.65; p<0.001). In this study, we observed a higher rate of early dissemination to the nasal mucosa than what was previously described. We suggest that an enhanced Th1 profile characterized by higher IL-12 is important for preventing dissemination of *L. braziliensis* to the mucosa. Further evaluation of parasite-related interactions with the host immunological response is necessary to elucidate the dissemination mechanisms of *Leishmania*.

## Introduction

Tegumentary leishmaniasis is a vector-borne disease with an incidence of approximately 0.7 to 1.2 million cases each year (World Health organization, 2021). The disease burden measured in disability-adjusted life years is higher than that of diseases such as leprosy, dengue fever and Chagas disease (Hotez et al., 2004). One of the main factors related to the negative impact of the disease is the possibility of sequelae related to the compromise of the nasal mucosa (Costa et al., 1987; Strazzulla et al., 2013). The presence of mucosal lesions is characteristic of American tegumentary leishmaniasis (ATL); it is estimated to occur in 3% to 5% of all patients with the disease in the Americas and is primarily caused by *Leishmania braziliensis* (Lessa et al., 2007).

Mucosal leishmaniasis (ML) is an outcome of ATL resulting from parasite dissemination from uncontained cutaneous leishmaniasis (CL) (Marsden et al., 1984; Amato et al., 2009). The presence of the parasite in the mucosa triggers an intense immune response dependent on macrophage activation (Da-Cruz et al., 2002; Carvalho et al., 2007; Conceição-Silva et al., 2018). This intense cellular reaction is responsible for the development of granulomatous lesions in the nasal septum and oropharyngeal structures (Conceição-Silva et al., 2018), reducing the quantity of parasites in late stages and diminishing the sensitivity of parasitological and molecular biology methods for diagnosis (Gomes et al., 2014). The natural history of ML shows that a possible paradox related to immunology exists. Although the presence of *Leishmania* in the mucosa over time stimulates an intense granulomatous response, earlier in disease progression, the parasite escapes from the host immunological response during dissemination periods to the mucosa (de Magalhães et al., 1986; Ávila et al., 2018; Martínez-López et al., 2018).

Early in the disease course, ML can coexist with active cutaneous lesions (Figueroa et al., 2009), but due to delayed access to healthcare, patients are normally diagnosed when the effects of destructive lesions, such as nasal septal perforation or destruction of facial architecture, have already occurred (Sampaio et al., 2019). The process begins with the dissemination of the parasite to the nasal mucosa, leading to microscopic and finally macroscopic changes (Amato et al., 2009). At first, lesions are oligosymptomatic and are not easily observed by the patient, which contributes to the delayed diagnosis (Boaventura et al., 2006). Later steps of disease development have been the focus of research in the field, limiting our understanding of the initial physio-pathogenic stages, including dissemination (Maretti-Mira et al., 2012; Ávila et al., 2018). *L. braziliensis* can be demonstrated in the nasal mucosa of patients with active cutaneous lesions (Figueroa et al., 2009; Romero et al., 2010; Canário et al., 2019), making these patients an interesting model to study early steps of disease development.

Here, we aimed to detect *L. braziliensis* DNA in the mucosa of ATL patients with active CL and to relate those findings with clinical and systemic immunological mediators related to the early dissemination (ED) of the parasite to the nasal mucosa.

## Method

We performed a cross-sectional study with patients attending from February 2017 to December 2020 the Leishmaniasis Clinic at the University Hospital of Brasilia, a referral service for leishmaniasis diagnosis and treatment in the Brazilian mid-western region. Initially, patients with active cutaneous lesions suggestive of leishmaniasis were screened for inclusion, and clinical and laboratory data were collected. After initial investigation, patients under immunosuppressive therapy or without a confirmed diagnosis of CL were excluded, and only patients with cutaneous lesions confirmed to be ATL were included. All CL cases were defined according to a reference standard based on the results of clinical evaluation, indirect immunofluorescense, direct skin exam, culture of skin aspirates in Novy-MacNeal-Nicolle medium and polymerase chain reactions of skin fragments as described elsewhere (Gomes et al., 2014; Gomes et al., 2014).

Patients with ATL early dissemination (CL-ED) were defined as active CL patients with the presence of *L. braziliensis* DNA in the nasal mucosa. Patients with negative testing were considered controls (CL-ED-Neg). In our study, all CL patients were examined by an assistant dermatologist with anterior rhinoscopy and oroscopy and were referenced for endoscopic examination of the ear, nose, and throat by an expert otorhinolaryngologist. Dermatological clinical examination also included measurement of cutaneous lesions using an adhesive ORC-9752 scale (Orc forensics, Oregon City, USA). In any patient with CL in whom an active mucous lesion was concomitantly identified, we applied Lessa’s classification as previously described: I, nodulation without ulcerations; II, superficial ulcerations; III, deep ulcerations; IV, septum perforation; and V, destruction of nasal architecture and altered facial structure (Lessa et al., 2012).

Anterior nasal swabs (Absorve™ sterile Sample collection swab, São Paulo, Brazil) were collected from all included patients. The swabs were rotated five times in each nasal fossa at the anterior septum and inferior turbinate head. DNA extraction from the nasal swab samples was performed using the PureLink Genomic DNA Kit (Invitrogen, Carlsbad, USA) according to the manufacturer’s protocol. Real-time polymerase chain reactions (RT-PCR) were performed with a TaqMan-specific probe for *L. braziliensis* detection (forward 5’-TGCTATAAAATCGTACCACCCGACA-3’, reverse 5’-GAACGGGGTTTCTGTATGCCATTT-3’), a probe for FAM (6-carboxyfluorescein; TTGCAGAACGCCCCTACCCAGAGGC), and TAMRA (6-carboxytetramethylrhodamine) on a QuantStudio 1 (Thermo Fisher Scientific, Waltham, USA), as described by Gomes et al. (2017). To evaluate circulating cytokine levels, plasma from each patient was isolated, and tumour necrosis factor (TNF)-α, interleukin (IL)-10, IL-1β, IL-8, IL-6 and IL-12p70 levels were measured using the Human Inflammatory Cytokine Cytometric Bead Array (Becton Dickinson, Franklin Lakes, USA) with a FACSVerse flow cytometer (Becton Dickinson).

For sample size calculation, we aimed at a power of 0.9 and an alpha of 0.05. The calculation was performed based on the expected levels of TNF-α extrapolated from a study by Da-Cruz et al (Da-Cruz et al., 1996), considering that the levels of this cytokine in subjects with ED would be similar to those observed in ML patients (231.4 ± 76.3 pg/ml) and that patients without ED would have a cytokine level comparable to that in CL patients (43.5 ± 8.2 pg/ml). We considered that approximately 11% (Gomes, 2014) of the CL patients would have ED and that 50% of the initially screened subjects would have CL confirmed after investigation. With these assumptions, we included 91 patients to have the desired power and alpha.

Regression imputation using the dependent variable and significant covariates was used to estimate three missing values of the total area of the lesions. The association between ED and clinical variables (sex, age, lesion evolution time, total area of the lesions, number of lesions, number of affected body segments, presence of mucosal symptoms and location of the lesions - hip or legs, arms, face or neck, and chest or abdomen) and cytokine levels was tested using two samples T tests for the parametrical variable “age” and Wilcoxon rank sum tests for all the other variables that were shown to be non-parametrical. All the variables associated with ED having p<0.25 were included in the multivariate model. Multivariate analysis was performed with Poisson regression with robust variance (Zou, 2004), and variables were chosen using the stepwise backward selection strategy. At each step, model improvement was evaluated by the Bayesian information criterion. Multicollinearity was also evaluated using the variance inflation factor (VIF). All statistical analyses were performed with Stata Statistical Software Release 16 (StataCorp LLC, College Station, USA). This study was approved by the institutional review board of the University of Brasilia (1.521.691), and subjects signed an informed consent form before inclusion.

## Results

**Figure 1:**
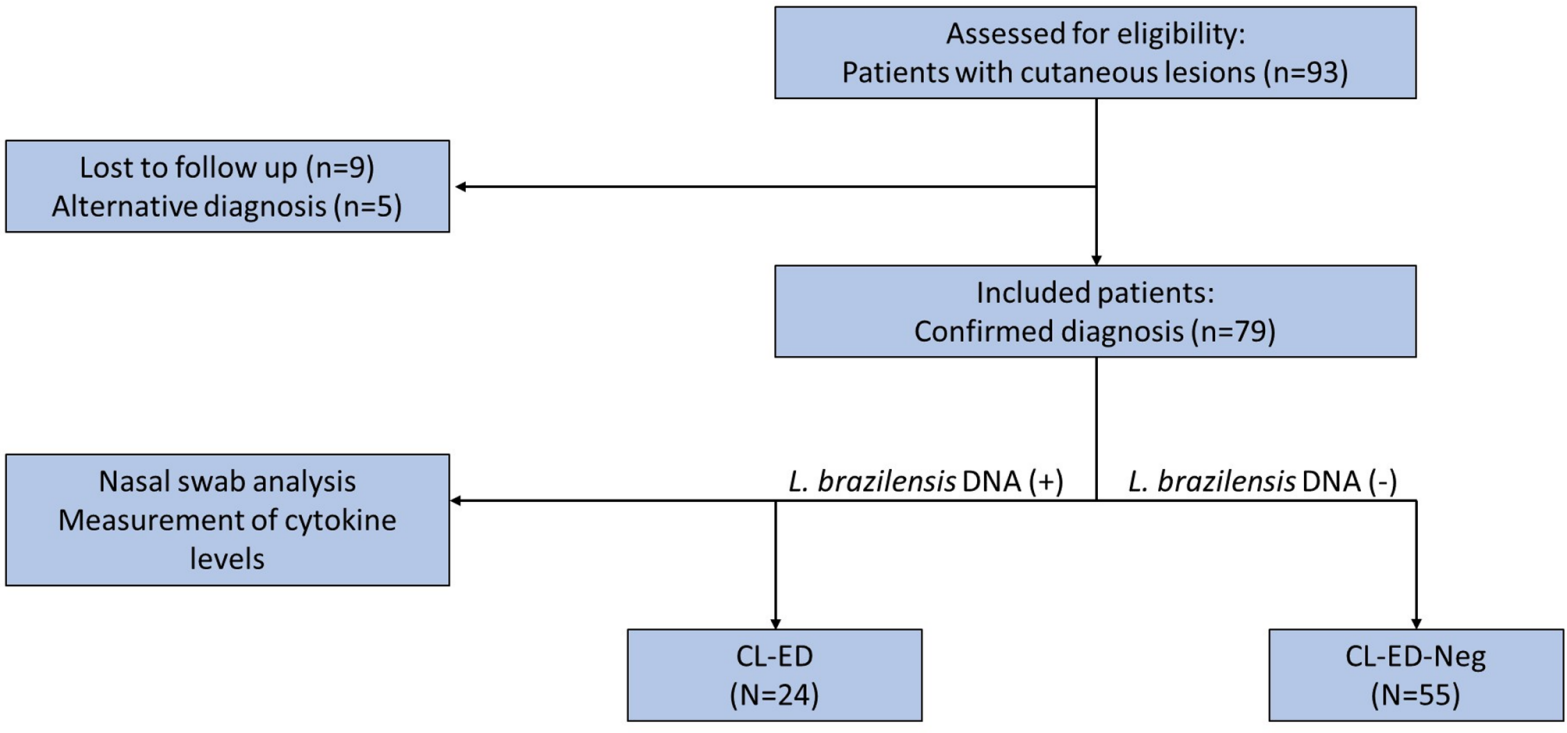
Flow chart showing eligible and included patients as well as the final group configuration. n, number of patients; CL-ED, cutaneous leishmaniasis with early dissemination to mucosa; CL-ED-Neg, cutaneous leishmaniasis without early dissemination to mucosa.

We recruited 93 patients, 14 of whom were excluded, nine for not having a leishmaniasis diagnosis and five for having an alternative diagnosis. The remaining 79 patients constituted our sample, with *L. braziliensis* DNA being identified in the nasal mucosa of 24 (30.3%) (**Figure 1**). The typical patient of our population was 41 years old, with a single lesion being approximately two months old on average. After otorhinolaryngological endoscopic examination, as expected, clinically active mucous lesions were found in only five patients in the CL-ED group (**Table 1**). In these patients, the lesions were early, not surpassing grade II in Lessa’s classification (Lessa et al., 2012) (**Table 1**). All patients were treated according to the national CL treatment protocol with meglumine antimoniate at a daily dose of 20 mg Sb5+/kg for 20 days.

**Table 1:** Description of early mucosal lesions of five patients with cutaneous leishmaniasis with concomitant mucous lesions and a positive result for *Leishmania braziliensis* DNA from nasal swabs.

ID	Clinical findings	Grade^†^
PCT1	Haematic crust on the nasal septum and swollen inferior turbinate mucosa	II
PCT2	Enanthem characterized by small red dot-like areas in the septal mucosa	I
PCT3	Infiltrative lesion at the base of the tongue	I
PCT4	Haematic crust in the left nasal septum region	II
PCT5	Aphthous lesion on the hard palate and nasal septum mucosa	II

^†^According to Lessa’s staging system: I, nodulation without ulcerations; II, superficial ulcerations; III, deep ulcerations; IV, septum perforation; V, destruction of nasal architecture and altered facial structure; ID, patient identification number.

Cytokine levels are disclosed in **Table 2** and were apparently similar between groups in the univariate analysis. In the univariate model, ED was significantly associated with a higher number of affected body segments (p=0.005) and a higher number of lesions (p= 0.003) (**Table 2**). Important variables in the univariate analysis were initially entered into the model (**Table 2**). High multicollinearity was found, and the TNF-α level was dropped from the model based on its VIF of 14.06 and its higher p value in the univariate model. After the selection of variables, the only variables that remained significant and thus were included in the model were IL-12 level (prevalence ratio (PR) =0.44; 95% confidence interval (CI): 0.21-0.94; p=0.034), IL-6 level (PR=1.001; 95%CI: 1.0005-1.002; p=0.002) and number of affected body segments (PR= 1.65; 95%CI: 1.36-2.01; p<0.001).

**Table 2:** Univariate analysis of possible risk factors for early dissemination of *Leishmania braziliensis* to the nasal mucosa.

Variables	CL-ED-Neg	CL-ED	p value
Age years (SD)^†^	42.6 (17.6)	38.4 (18.4)	0.17
Time since disease onset, median (IQR)	2 (2)	2 (1.5)	0.87
Number of lesions (SD)^†^	1 (1)	1 (4.5)	0.003
Number of affected body segments, median (IQR)^†^	1 (0)	1 (1)	0.005
Total area of the lesions (cm^2^)^†^	11.94 (12.22)	17.56 (16.07)	0.097
Sex			
Male (%)	35 (63.6)	15 (62.5)	1
Female (%)	20 (36.6)	9 (37.5)	
Mucosal symptoms (%)	13 (26.3)	7 (29.17)	0.580
Cutaneous lesions on the legs and hip (%)	22 (40)	12 (50)	0.464
Cutaneous lesions on the arms (%)^†^	16 (29)	11 (45.8)	0.198
Cutaneous lesions on the thorax and abdomen (%)^†^	3 (5.4)	5 (20.8)	0.051
Cutaneous lesions on the head and neck (%)^†^	5 (9)	6 (25)	0.080
Cytokine levels (pg/ml)			
IL-12 (SD)^†^	4.09 (2.8)	3.19 (0.2)	0.170
TNF-α (SD)^†^	7.93 (11.7)	44.79 (195.9)	0.240
IL-10 (SD)	3.21 (2.1)	2.54 (0.7)	0.390
IL-6 (SD)^†^	15.98 (41.6)	41.41 (171)	0.170
IL-1β (SD)^†^	6.12 (1.3)	6.06 (2.2)	0.160
IL-8 (SD)	243.2 (576.6)	421.08 (1,216.3)	0.850

TNF, tumour necrosis factor; IL, interleukin; SD, standard deviation; IQR, interquartile range. ^†^Variables initially entered in the model. CL-ED, cutaneous leishmaniasis with early dissemination to mucosa; CL-ED-Neg, cutaneous leishmaniasis without early dissemination to mucosa.

## Discussion

In this study, we observed that 30.3% of patients with active cutaneous ulcers had molecular evidence of the presence of *L. braziliensis* in the nasal mucosa. In another study from an *L. braziliensis* endemic area, Canário et al. found parasite DNA in the mucosa of 7.8% CL subjects with clinically healthy nasal mucosa (Canário et al., 2019). This difference may be partially explained by the distinct inclusion criteria of the studies, since we did not exclude patients with CL if they were shown to have simultaneous early mucosal lesions.

In addition, all mucosal lesions found were early, not surpassing grade II of V in Lessa’s classification (Lessa et al., 2012). Conversely, other studies have shown that by the time of diagnosis, approximately 60% of active ML cases are classified as grade III or greater (Lessa et al., 2012; de Lima et al., 2021). Thus, the early diagnosis of mucosal lesions in this study was associated with shorter disease duration, three months on average, and consequently less severe disease. Our data thus support the proposition by Boaventura et al. that earlier diagnosis may be made if patients with cutaneous lesions are subjected to systematic otorhinolaryngological examination (Boaventura et al., 2006). On the other hand, the proportion of patients with evidence of ED to the nasal mucosa in our study was lower than those found in Colombia (45%-58%) (Figueroa et al., 2009; Martínez-Valencia et al., 2017). It is possible that this difference between studies is partially explained by the *Leishmania* species responsible, since in Colombia, most of the isolates are from *Leishmania panamensis* (Martínez-Valencia et al., 2017), and most mucosal lesions (61%) due to this species occur simultaneously with the active ulcer (Osorio et al., 1998).

Clinical lesions on the mucosa can be difficult to access, and early lesions can lead to only discolouration of mucosa long before the beginning of mucous-specific symptoms (Nishino et al., 1986; Oliveira et al., 1995; Silveira, 2019). Consequently, these lesions can sometimes only be found by expert examiners with the use of special equipment and magnification. Once ML requires longer courses of treatment, the recognition of early mucous lesions is paramount for achieving satisfactory cure (Ministério da Saúde do Brasil SDVES and Departamento de Vigilância Epidemiológica, 2017).

Dissemination of the parasite from an initial cutaneous lesion is assumed to be the main mechanism of ML development (Marsden et al., 1984; Amato et al., 2009). Although conceivable, multiple infections are unlikely to be the culprit for this form. Parasite similarity occurs between cutaneous and mucosal lesions of the same patient, with the parasite genetic variability in these patient lesions being less prominent than the variability between different patients, as shown in an *L. braziliensis* kDNA signature study (Oliveira et al., 2013). Another possibility is that the parasite disseminates later in the disease process. It has been suggested that amastigotes from ML patients have a decreased ability to be internalized and grow more slowly than those from CL patients (Gomes et al., 2016) and that stationary phase promastigotes from ML are more resistant to nitric oxide (Ávila et al., 2018). These findings suggest that ML parasites are transmitted by vectors as promastigotes that are initially more resistant and more likely to disseminate (Ávila et al., 2018). Additionally, the protective immune responses against *L. braziliensis* are weaker in the early disease process (Unger et al., 2009). Thus, parasite and host immune factors make it more likely that early dissemination is the essential step leading to the development of ML in most cases.

In line with the pool of evidence found in the literature, our univariate analysis showed that the number of lesions and the number of affected body segments were associated with ED of *L. braziliensis* to the nasal mucosa. A greater number of lesions was also found to be associated with ED in another study (Maretti-Mira et al., 2012). Interestingly, the clinical risk factors we found were the same as those found in previous studies (Marsden et al., 1984; Cuentas et al., 1984; Turetz et al., 2002), suggesting a predictive relationship between ED and the possible development of ML. The confirmation of this relationship, however, would require a study with long-term follow-up, since many patients commonly have cutaneous lesions a long time before the development of ML (with 37.1% having lesions more than 5 years earlier and 5.7% having lesions more than 15 years earlier according to one classical study (Marsden et al., 1984).

ML is characterized by a marked inflammatory infiltrate that is responsible for maintaining autoaggression in the presence of few or no parasites (de Magalhães et al., 1986). The inflammatory process is driven by excess proinflammatory mediators such as TNF-α and IFN-γ that are unchecked by regulatory cytokines such as IL-10 and TGF-β (Bacellar et al., 2002; Campos et al., 2018). TNF-α is the most studied mediator (Castes et al., 1993; Cabrera et al., 1995; Da-Cruz et al., 1996; Ribeiro-de-Jesus et al., 1998), but other inflammatory cytokines, such as IL-6 (Castellucci et al., 2006), IL-1β (Moreira et al., 2017) and IL-8 (Vargas-Inchaustegui et al., 2009), may also play a role. In the classical paradigm framework, the development of ML is related to a Th1 phenotype characterized by CD4+ T lymphocytes expressing IFN-γ. In this context, IL-12 is an inducer, and IL-10 is an opposing cytokine (Ribeiro-de-Jesus et al., 1998; Silveira, 2019). However, the same inflammatory mediators linked to protection also play a role in tissue destruction associated with ML (Hartley et al., 2014). Some authors have suggested that this apparent paradox may be associated with the dynamics of inflammatory cytokine expression in relation to disease evolution (Ribeiro-de-Jesus et al., 1998; Unger et al., 2009), with early diminished IFN-γ and TNF-α expression being associated with worse prognosis (Unger et al., 2009). We investigated the association of key cytokines with the ED of *L. braziliensis* to the nasal mucosa to better understand the role of the early immune response in the development of ML. To account for possible confounding clinical variables, we opted to use statistical multivariate modelling to assess the association between cytokine levels and the outcome. Higher IL-6 levels were significantly associated with ED in our final multivariate model (PR=1.001; p=0.002). Although IL-6 has been implicated in the development of mucosal disease (Castellucci et al., 2006), in our study, the effect size (PR=1.001; p=0.002) was too low for the result to be considered of physio-pathological significance, especially considering the effect sizes of the other independent variables included in the model.

IL-12 was a protective mediator against ED of *L. braziliensis* to the nasal mucosa (PR=0.44; p=0.034; 95%CI: 0.21-0.94) in our final multivariate model. IL-12 is a cytokine classically associated with the development of the Th1 cytokine response (O'Garra and Vieira, 2007) and is in the proinflammatory cytokine milieu associated with the development of ML (Ribeiro-de-Jesus et al., 1998; Silveira, 2019). This protective role of IL-12 seems contradictory since most studies have linked the development of ML with the proinflammatory Th1 response (Castes et al., 1993; Da-Cruz et al., 2002; Bacellar et al., 2002). However, this finding may be explained by the scarcity of studies that address the early immunopathological steps leading to ML development. In a transcriptomic study with ten patients with CL, five of whom later developed ML, Maretti-Mira et al. suggested that patients prone to mucosal disease have an insufficient or delayed development of immune response (Maretti-Mira et al., 2012). In another study by Gomes et al, IFN-γ knockout (KO) mice were infected with amastigotes from patients with ML and CL. Interestingly, in mice infected with ML-derived amastigotes, cutaneous lesions appeared later, but a greater number of parasites were observed in the spleen (Gomes et al., 2016). The same group later showed that the stationary-phase promastigotes derived from ML are more resistant to killing by nitric oxide (NO) and reactive oxygen species than are the CL-derived promastigotes. On the other hand, a lower resistance of ML amastigotes to NO was observed. Altogether, the findings of these two studies suggest that earlier in infection, ML parasites have a weaker capacity to stimulate the immune response at the site of infection but a greater ability to disseminate (Ávila et al., 2018). The delayed development of a protective immune response may facilitate parasite dissemination, and chronic systemic exposure to *Leishmania* antigens could in turn lead to the exacerbated inflammatory immune response associated with mucosal disease. This idea is in accordance with the finding that in cured ML patients, the duration of active disease is positively associated with antigen-induced production of IFN-γ but negatively associated with IL-10 production (Nogueira et al., 2014).

It is important to state that although this study represents the evaluation of a representative population the presence of untested confounders is always a limitation of clinical observational studies. This is especially true for the study of host-parasite immunological interaction. The present results show that future experiments are feasible and paramount for the study of dissemination mechanisms present in ML cases. The use of more complex laboratory methods, the search for a wider panel of mediators, a wider profile of clinical characteristics and the investigation of *Leishmania* RNA virus associations are important factors that must be searched in future protocols and that may significantly influence the clinical presentation of CL and ML.

Our study shows that the Th1 response, characterized by higher IL-12 levels, possibly helps limit the disease to the cutaneous site of infection, preventing dissemination to the nasal mucosa. We hypothesize that during the cutaneous phase of the disease, an insufficient rather than exacerbated immune response is responsible for the dissemination of the parasite and later development of mucous disease. These findings are, however, of exploratory nature and deserve to be more thoroughly investigated in other studies. Our study also highlights the importance of a detailed search for mucous commitment in CL to avoid insufficient treatment.

## Data Availability Statement

The raw data supporting the conclusions of this article will be made available by the authors, without undue reservation.

## Ethics Statement

The studies involving human participants were reviewed and approved by Comitê de Ética em Pesquisa da Faculdade de Medicina da Universidade de Brasília - UnB. Written informed consent to participate in this study was provided by the participants’ legal guardian/next of kin.

## Author Contributions

DB: conception, design, data acquisition, analysis, interpretation of data, drafting, final approval. OT: data acquisition, analysis, interpretation of data. CA: data acquisition, analysis, interpretation of data. GF: data acquisition, analysis and interpretation. SS: data acquisition and analysis. BR: data acquisition and analysis. CG: conception, design, data acquisition, analysis, interpretation of data, drafting, final approval, supervision. RS: conception, design, data acquisition, analysis, interpretation of data, drafting, final approval, supervision. All authors contributed to the article and approved the submitted version.

## Funding

This study was financed in part by the Coordenação de Aperfeiçoamento de Pessoal de Nível Superior - Brasil (CAPES) - Finance Code 001. This work was also supported by grants - 16854.78.40785.2604/2017 and 0193.001447/2016- from Fundação de Apoio à Pesquisa do Distrito Federal (FAP-DF), and from Brazilian Society of Dermatology through FUNADERM.

## Conflict of Interest

The authors declare that the research was conducted in the absence of any commercial or financial relationships that could be construed as a potential conflict of interest.

## Publisher’s Note

All claims expressed in this article are solely those of the authors and do not necessarily represent those of their affiliated organizations, or those of the publisher, the editors and the reviewers. Any product that may be evaluated in this article, or claim that may be made by its manufacturer, is not guaranteed or endorsed by the publisher.
